# (*Z*)-1-(4-Methyl­phen­yl)-2-(phenyl­sulfon­yl)ethanone oxime

**DOI:** 10.1107/S1600536812030346

**Published:** 2012-07-07

**Authors:** Hoong-Kun Fun, Tze Shyang Chia, Khalid A. Al-Rashood, Hatem A. Abdel-Aziz

**Affiliations:** a X-ray Crystallography Unit, School of Physics, Universiti Sains Malaysia, 11800 USM, Penang, Malaysia; b Department of Pharmaceutical Chemistry, College of Pharmacy, King Saud University, PO Box 2457, Riyadh 11451, Saudi Arabia

## Abstract

The mol­ecule of the title compound, C_15_H_15_NO_3_S, has a twisted U-shaped conformation: the twist occurs at the central C—S(=O)_2_—C—C—C unit and the benzene ring makes a dihedral angle of 28.74 (7)° with the phenyl ring. The S—C—C=N torsion angle is −88.95 (13)°. In the crystal, inversion dimers linked by pairs of O—H⋯N hydrogen bonds generate *R*_2_^2^(6) loops, and C—H⋯O hydrogen bonds connect the dimers into a three-dimensional network.

## Related literature

For the biological activity of aryl­sulphones, see: Stephens *et al.* (2001 ▶); Abdel-Aziz *et al.* (2010 ▶). For graph-set notation of hydrogen bonds, see: Bernstein *et al.* (1995 ▶). For the stability of the temperature controller used for the data collection, see: Cosier & Glazer (1986 ▶).
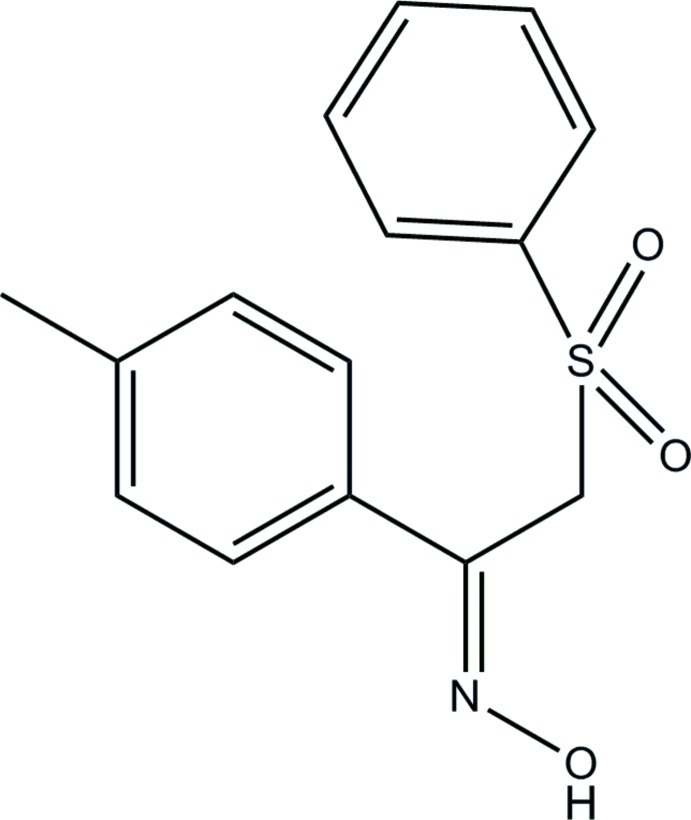


## Experimental

### 

#### Crystal data

C_15_H_15_NO_3_S
*M* *_r_* = 289.34
Monoclinic, 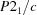

*a* = 5.2305 (3) Å
*b* = 17.6073 (11) Å
*c* = 15.6578 (10) Å
β = 103.782 (2)°
*V* = 1400.49 (15) Å^3^
*Z* = 4
Mo *K*α radiation
μ = 0.24 mm^−1^
*T* = 100 K
0.29 × 0.09 × 0.06 mm

#### Data collection

Bruker APEX DUO CCD diffractometer
Absorption correction: multi-scan (*SADABS*; Bruker, 2009 ▶) *T*_min_ = 0.934, *T*_max_ = 0.985
16727 measured reflections
4349 independent reflections
3578 reflections with *I* > 2σ(*I*)
*R*_int_ = 0.030

#### Refinement

*R*[*F* ^2^ > 2σ(*F* ^2^)] = 0.037
*wR*(*F* ^2^) = 0.105
*S* = 1.03
4349 reflections
186 parameters
H atoms treated by a mixture of independent and constrained refinement
Δρ_max_ = 0.42 e Å^−3^
Δρ_min_ = −0.33 e Å^−3^

### 

Data collection: *APEX2* (Bruker, 2009 ▶); cell refinement: *SAINT* (Bruker, 2009 ▶); data reduction: *SAINT*; program(s) used to solve structure: *SHELXTL* (Sheldrick, 2008 ▶); program(s) used to refine structure: *SHELXTL*; molecular graphics: *SHELXTL*; software used to prepare material for publication: *SHELXTL* and *PLATON* (Spek, 2009 ▶).

## Supplementary Material

Crystal structure: contains datablock(s) global, I. DOI: 10.1107/S1600536812030346/hb6884sup1.cif

Structure factors: contains datablock(s) I. DOI: 10.1107/S1600536812030346/hb6884Isup2.hkl

Supplementary material file. DOI: 10.1107/S1600536812030346/hb6884Isup3.cml

Additional supplementary materials:  crystallographic information; 3D view; checkCIF report

## Figures and Tables

**Table 1:** Hydrogen-bond geometry (Å, °)

*D*—H⋯*A*	*D*—H	H⋯*A*	*D*⋯*A*	*D*—H⋯*A*
O3—H1O3⋯N1^i^	0.97 (2)	1.88 (2)	2.7819 (15)	153.8 (18)
C2—H2*A*⋯O2^ii^	0.93	2.53	3.2413 (18)	134
C3—H3*A*⋯O1^iii^	0.93	2.56	3.4720 (17)	169
C7—H7*A*⋯O1^iv^	0.97	2.34	3.2313 (14)	153
C15—H15*B*⋯O2^v^	0.96	2.54	3.426 (2)	154

Symmetry codes: (i) 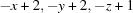; (ii) 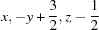; (iii) 
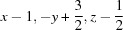
; (iv) 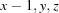; (v) 
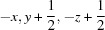
.

**Fig. 1.**
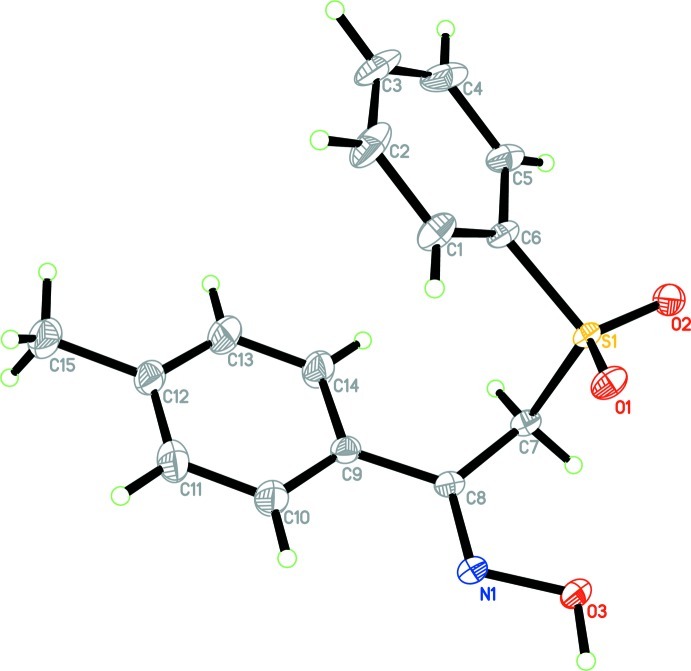
The molecular structure of the title compound with 50% probability displacement ellipsoids.

**Fig. 2.**
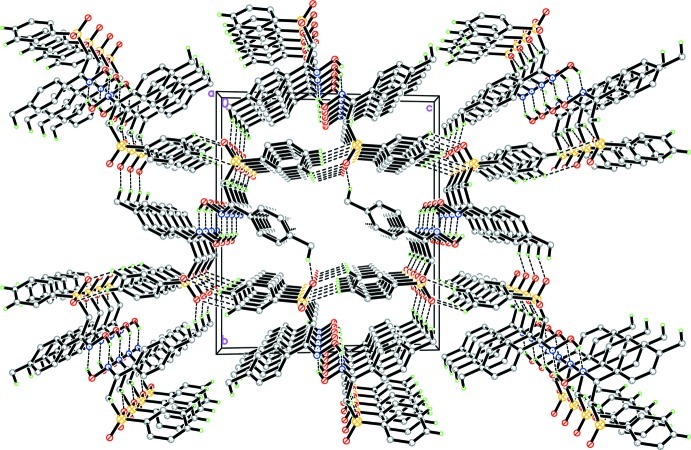
The crystal packing of the title compound. The dashed lines represent the hydrogen bonds. For clarity sake, hydrogen atoms not involved in hydrogen bonding have been omitted.

## supplementary crystallographic information

### Comment

Arylsulphones possess interesting biological activities (Stephens *et al.*, 2001; Abdel-Aziz *et al.*, 2010). As part of our studies in this area, we report herein the crystal structure of the title compound, (*Z*)-1-(4-methylphenyl)-2-(phenylsulfonyl)ethanone oxime.

The molecular structure of the title compound is shown in Fig. 1. The molecule adopts a twisted U-shaped conformation. The twist occurs at the central C6–S1–C7–C8–C9 unit and the C1–C6 benzene ring makes a dihedral angle of 28.74 (7)° with the C9–C14 phenyl ring.

In the crystal (Fig. 2), the molecules are linked by O3—H1O3···N1, C2—H2A···O2, C3—H3A···O1, C7—H7A···O1 and C15—H15B···O2 hydrogen bonds (Table 1) into a three-dimensional network.

### Experimental

A mixture of 2-(phenylsulfonyl)-1-*p*-tolylethanone (0.274 g, 1 mmol), hydroxylamine hydrochloride (0.11 g, 1.5 mmol) and anhydrous sodium acetate (0.123 g, 1.5 mmol) in ethanol (50 ml) was refluxed for 1 h, then left to cool. The reaction mixture was poured into cold water and the solid product was filtered off, washed with water, dried and finally recrystallized from ethanol to afford the title compound as yellow needles.

### Refinement

Atom H1O3 was located in a difference fourier map and refined freely [O3—H1O3 = 0.96 (2) Å]. The remaining H atoms were positioned geometrically [C—H = 0.93, 0.96 and 0.97 Å] and refined using a riding model with *U*_iso_(H) = 1.2 or 1.5*U*_eq_(C). A rotating group model was applied to the methyl group.

### Crystal data

**Table:**

C_15_H_15_NO_3_S	*F*(000) = 608
*M**_r_* = 289.34	*D*_x_ = 1.372 Mg m^−^^3^
Monoclinic, *P*2_1_/*c*	Mo *K*α radiation, λ = 0.71073 Å
Hall symbol: -P 2ybc	Cell parameters from 5520 reflections
*a* = 5.2305 (3) Å	θ = 2.3–30.7°
*b* = 17.6073 (11) Å	µ = 0.24 mm^−^^1^
*c* = 15.6578 (10) Å	*T* = 100 K
β = 103.782 (2)°	Needle, yellow
*V* = 1400.49 (15) Å^3^	0.29 × 0.09 × 0.06 mm
*Z* = 4	

### Data collection

**Table:**

Bruker APEX DUO CCD diffractometer	4349 independent reflections
Radiation source: fine-focus sealed tube	3578 reflections with *I* > 2σ(*I*)
Graphite monochromator	*R*_int_ = 0.030
φ and ω scans	θ_max_ = 30.8°, θ_min_ = 1.8°
Absorption correction: multi-scan (*SADABS*; Bruker, 2009)	*h* = −7→7
*T*_min_ = 0.934, *T*_max_ = 0.985	*k* = −21→25
16727 measured reflections	*l* = −16→22

### Refinement

**Table:**

Refinement on *F*^2^	Primary atom site location: structure-invariant direct methods
Least-squares matrix: full	Secondary atom site location: difference Fourier map
*R*[*F*^2^ > 2σ(*F*^2^)] = 0.037	Hydrogen site location: inferred from neighbouring sites
*wR*(*F*^2^) = 0.105	H atoms treated by a mixture of independent and constrained refinement
*S* = 1.03	*w* = 1/[σ^2^(*F*_o_^2^) + (0.0504*P*)^2^ + 0.6281*P*] where *P* = (*F*_o_^2^ + 2*F*_c_^2^)/3
4349 reflections	(Δ/σ)_max_ = 0.001
186 parameters	Δρ_max_ = 0.42 e Å^−^^3^
0 restraints	Δρ_min_ = −0.33 e Å^−^^3^

### Special details

**Table:**

Experimental. The crystal was placed in the cold stream of an Oxford Cryosystems Cobra open-flow nitrogen cryostat (Cosier & Glazer, 1986) operating at 100.0 (1) K.
Geometry. All e.s.d.'s (except the e.s.d. in the dihedral angle between two l.s. planes) are estimated using the full covariance matrix. The cell e.s.d.'s are taken into account individually in the estimation of e.s.d.'s in distances, angles and torsion angles; correlations between e.s.d.'s in cell parameters are only used when they are defined by crystal symmetry. An approximate (isotropic) treatment of cell e.s.d.'s is used for estimating e.s.d.'s involving l.s. planes.
Refinement. Refinement of *F*^2^ against ALL reflections. The weighted *R*-factor *wR* and goodness of fit *S* are based on *F*^2^, conventional *R*-factors *R* are based on *F*, with *F* set to zero for negative *F*^2^. The threshold expression of *F*^2^ > σ(*F*^2^) is used only for calculating *R*-factors(gt) *etc*. and is not relevant to the choice of reflections for refinement. *R*-factors based on *F*^2^ are statistically about twice as large as those based on *F*, and *R*-factors based on ALL data will be even larger.

### Fractional atomic coordinates and isotropic or equivalent isotropic displacement parameters (Å^2^)

**Table:**

	*x*	*y*	*z*	*U*_iso_*/*U*_eq_	
S1	0.56144 (5)	0.782145 (17)	0.384686 (18)	0.01353 (8)	
O1	0.83354 (16)	0.79802 (6)	0.38609 (6)	0.01848 (19)	
O2	0.50126 (19)	0.71628 (5)	0.43065 (6)	0.0208 (2)	
O3	0.86263 (19)	0.92446 (6)	0.52121 (6)	0.0218 (2)	
N1	0.7376 (2)	0.96756 (6)	0.44763 (7)	0.0177 (2)	
C1	0.4951 (3)	0.81035 (9)	0.20998 (9)	0.0239 (3)	
H1A	0.6457	0.8401	0.2263	0.029*	
C2	0.3699 (3)	0.80091 (11)	0.12183 (9)	0.0319 (3)	
H2A	0.4350	0.8249	0.0785	0.038*	
C3	0.1472 (3)	0.75546 (11)	0.09865 (10)	0.0329 (4)	
H3A	0.0655	0.7485	0.0396	0.039*	
C4	0.0457 (3)	0.72046 (10)	0.16220 (10)	0.0303 (3)	
H4A	−0.1042	0.6905	0.1456	0.036*	
C5	0.1662 (2)	0.72973 (8)	0.25099 (9)	0.0214 (3)	
H5A	0.0981	0.7066	0.2942	0.026*	
C6	0.3915 (2)	0.77457 (7)	0.27336 (8)	0.0164 (2)	
C7	0.4295 (2)	0.86214 (7)	0.43094 (8)	0.0154 (2)	
H7A	0.2388	0.8605	0.4130	0.018*	
H7B	0.4803	0.8586	0.4946	0.018*	
C8	0.5234 (2)	0.93671 (7)	0.40293 (8)	0.0153 (2)	
C9	0.3773 (2)	0.97524 (7)	0.32203 (8)	0.0168 (2)	
C10	0.4854 (3)	1.03660 (9)	0.28673 (11)	0.0303 (3)	
H10A	0.6487	1.0555	0.3160	0.036*	
C11	0.3522 (3)	1.06969 (10)	0.20849 (11)	0.0349 (4)	
H11A	0.4286	1.1106	0.1863	0.042*	
C12	0.1092 (3)	1.04366 (8)	0.16238 (9)	0.0255 (3)	
C13	0.0000 (3)	0.98351 (10)	0.19822 (11)	0.0325 (3)	
H13A	−0.1644	0.9652	0.1690	0.039*	
C14	0.1302 (3)	0.94978 (9)	0.27687 (10)	0.0283 (3)	
H14A	0.0512	0.9097	0.2996	0.034*	
C15	−0.0305 (4)	1.07962 (10)	0.07609 (11)	0.0353 (4)	
H15A	0.0951	1.0911	0.0422	0.053*	
H15B	−0.1154	1.1256	0.0875	0.053*	
H15C	−0.1600	1.0450	0.0439	0.053*	
H1O3	1.021 (4)	0.9523 (12)	0.5474 (13)	0.040 (5)*	

### Atomic displacement parameters (Å^2^)

**Table:**

	*U*^11^	*U*^22^	*U*^33^	*U*^12^	*U*^13^	*U*^23^
S1	0.01289 (12)	0.01713 (15)	0.00974 (13)	−0.00179 (9)	0.00102 (9)	−0.00080 (10)
O1	0.0124 (3)	0.0265 (5)	0.0157 (4)	−0.0008 (3)	0.0017 (3)	−0.0018 (4)
O2	0.0274 (5)	0.0191 (5)	0.0158 (4)	−0.0032 (4)	0.0050 (3)	0.0019 (4)
O3	0.0217 (4)	0.0216 (5)	0.0176 (5)	−0.0041 (4)	−0.0045 (3)	0.0019 (4)
N1	0.0169 (4)	0.0175 (5)	0.0162 (5)	−0.0011 (4)	−0.0014 (4)	−0.0007 (4)
C1	0.0232 (6)	0.0341 (8)	0.0139 (6)	−0.0006 (5)	0.0036 (5)	−0.0002 (5)
C2	0.0334 (7)	0.0499 (10)	0.0114 (6)	0.0084 (7)	0.0033 (5)	0.0008 (6)
C3	0.0275 (6)	0.0521 (10)	0.0142 (6)	0.0124 (7)	−0.0047 (5)	−0.0123 (7)
C4	0.0181 (6)	0.0409 (9)	0.0274 (8)	0.0022 (5)	−0.0037 (5)	−0.0171 (7)
C5	0.0159 (5)	0.0267 (7)	0.0206 (6)	−0.0008 (5)	0.0019 (4)	−0.0079 (5)
C6	0.0148 (5)	0.0219 (6)	0.0113 (5)	0.0005 (4)	0.0005 (4)	−0.0032 (5)
C7	0.0143 (4)	0.0191 (6)	0.0128 (5)	−0.0023 (4)	0.0033 (4)	−0.0019 (4)
C8	0.0140 (5)	0.0163 (6)	0.0150 (5)	−0.0008 (4)	0.0025 (4)	−0.0029 (4)
C9	0.0170 (5)	0.0163 (6)	0.0161 (6)	0.0015 (4)	0.0018 (4)	−0.0025 (5)
C10	0.0296 (7)	0.0260 (8)	0.0290 (8)	−0.0092 (6)	−0.0057 (6)	0.0067 (6)
C11	0.0402 (8)	0.0265 (8)	0.0327 (8)	−0.0056 (6)	−0.0021 (6)	0.0115 (7)
C12	0.0322 (7)	0.0206 (7)	0.0201 (6)	0.0107 (5)	−0.0008 (5)	−0.0014 (5)
C13	0.0256 (6)	0.0333 (8)	0.0303 (8)	0.0002 (6)	−0.0099 (5)	0.0050 (7)
C14	0.0209 (6)	0.0286 (8)	0.0299 (8)	−0.0052 (5)	−0.0047 (5)	0.0072 (6)
C15	0.0474 (9)	0.0279 (8)	0.0243 (8)	0.0146 (7)	−0.0037 (6)	0.0014 (6)

### Geometric parameters (Å, º)

**Table:**

S1—O2	1.4386 (10)	C7—C8	1.5034 (17)
S1—O1	1.4454 (9)	C7—H7A	0.9700
S1—C6	1.7630 (12)	C7—H7B	0.9700
S1—C7	1.7951 (13)	C8—C9	1.4791 (17)
O3—N1	1.4042 (14)	C9—C14	1.3913 (17)
O3—H1O3	0.96 (2)	C9—C10	1.393 (2)
N1—C8	1.2910 (15)	C10—C11	1.385 (2)
C1—C2	1.3896 (19)	C10—H10A	0.9300
C1—C6	1.3898 (18)	C11—C12	1.382 (2)
C1—H1A	0.9300	C11—H11A	0.9300
C2—C3	1.388 (2)	C12—C13	1.384 (2)
C2—H2A	0.9300	C12—C15	1.513 (2)
C3—C4	1.380 (2)	C13—C14	1.390 (2)
C3—H3A	0.9300	C13—H13A	0.9300
C4—C5	1.3925 (19)	C14—H14A	0.9300
C4—H4A	0.9300	C15—H15A	0.9600
C5—C6	1.3918 (17)	C15—H15B	0.9600
C5—H5A	0.9300	C15—H15C	0.9600
			
O2—S1—O1	118.71 (6)	S1—C7—H7B	109.1
O2—S1—C6	108.34 (6)	H7A—C7—H7B	107.8
O1—S1—C6	107.00 (6)	N1—C8—C9	118.42 (12)
O2—S1—C7	106.24 (6)	N1—C8—C7	120.73 (11)
O1—S1—C7	108.29 (6)	C9—C8—C7	120.82 (10)
C6—S1—C7	107.85 (6)	C14—C9—C10	117.63 (12)
N1—O3—H1O3	105.0 (12)	C14—C9—C8	121.03 (12)
C8—N1—O3	113.06 (11)	C10—C9—C8	121.31 (11)
C2—C1—C6	118.90 (14)	C11—C10—C9	120.72 (13)
C2—C1—H1A	120.6	C11—C10—H10A	119.6
C6—C1—H1A	120.6	C9—C10—H10A	119.6
C3—C2—C1	119.75 (15)	C12—C11—C10	121.92 (15)
C3—C2—H2A	120.1	C12—C11—H11A	119.0
C1—C2—H2A	120.1	C10—C11—H11A	119.0
C4—C3—C2	120.79 (13)	C11—C12—C13	117.32 (13)
C4—C3—H3A	119.6	C11—C12—C15	121.08 (15)
C2—C3—H3A	119.6	C13—C12—C15	121.60 (14)
C3—C4—C5	120.48 (14)	C12—C13—C14	121.57 (14)
C3—C4—H4A	119.8	C12—C13—H13A	119.2
C5—C4—H4A	119.8	C14—C13—H13A	119.2
C6—C5—C4	118.19 (14)	C13—C14—C9	120.81 (14)
C6—C5—H5A	120.9	C13—C14—H14A	119.6
C4—C5—H5A	120.9	C9—C14—H14A	119.6
C1—C6—C5	121.87 (12)	C12—C15—H15A	109.5
C1—C6—S1	118.76 (10)	C12—C15—H15B	109.5
C5—C6—S1	119.27 (10)	H15A—C15—H15B	109.5
C8—C7—S1	112.57 (8)	C12—C15—H15C	109.5
C8—C7—H7A	109.1	H15A—C15—H15C	109.5
S1—C7—H7A	109.1	H15B—C15—H15C	109.5
C8—C7—H7B	109.1		
			
C6—C1—C2—C3	−0.8 (2)	O3—N1—C8—C7	0.08 (16)
C1—C2—C3—C4	1.1 (2)	S1—C7—C8—N1	−88.95 (13)
C2—C3—C4—C5	−0.4 (2)	S1—C7—C8—C9	89.10 (12)
C3—C4—C5—C6	−0.5 (2)	N1—C8—C9—C14	−172.31 (13)
C2—C1—C6—C5	−0.1 (2)	C7—C8—C9—C14	9.59 (19)
C2—C1—C6—S1	176.35 (12)	N1—C8—C9—C10	9.84 (19)
C4—C5—C6—C1	0.7 (2)	C7—C8—C9—C10	−168.25 (13)
C4—C5—C6—S1	−175.68 (11)	C14—C9—C10—C11	−1.3 (2)
O2—S1—C6—C1	−152.81 (11)	C8—C9—C10—C11	176.65 (15)
O1—S1—C6—C1	−23.72 (13)	C9—C10—C11—C12	−0.1 (3)
C7—S1—C6—C1	92.59 (11)	C10—C11—C12—C13	1.1 (3)
O2—S1—C6—C5	23.70 (12)	C10—C11—C12—C15	−178.77 (16)
O1—S1—C6—C5	152.79 (10)	C11—C12—C13—C14	−0.8 (3)
C7—S1—C6—C5	−90.91 (11)	C15—C12—C13—C14	179.06 (15)
O2—S1—C7—C8	168.32 (8)	C12—C13—C14—C9	−0.5 (3)
O1—S1—C7—C8	39.77 (10)	C10—C9—C14—C13	1.5 (2)
C6—S1—C7—C8	−75.70 (9)	C8—C9—C14—C13	−176.38 (14)
O3—N1—C8—C9	−178.02 (10)		

### Hydrogen-bond geometry (Å, º)

**Table:**

*D*—H···*A*	*D*—H	H···*A*	*D*···*A*	*D*—H···*A*
O3—H1*O*3···N1^i^	0.97 (2)	1.88 (2)	2.7819 (15)	153.8 (18)
C2—H2*A*···O2^ii^	0.93	2.53	3.2413 (18)	134
C3—H3*A*···O1^iii^	0.93	2.56	3.4720 (17)	169
C7—H7*A*···O1^iv^	0.97	2.34	3.2313 (14)	153
C15—H15*B*···O2^v^	0.96	2.54	3.426 (2)	154

Symmetry codes: (i) −*x*+2, −*y*+2, −*z*+1; (ii) *x*, −*y*+3/2, *z*−1/2; (iii) *x*−1, −*y*+3/2, *z*−1/2; (iv) *x*−1, *y*, *z*; (v) −*x*, *y*+1/2, −*z*+1/2.
